# Child abuse knowledge and attitudes among dental and oral health therapists in Aotearoa New Zealand: a cross-sectional study

**DOI:** 10.1186/s12913-022-08907-1

**Published:** 2022-12-10

**Authors:** Heuiwon Han, Amanda B. Lees, Zac Morse, Jane Koziol-McLain

**Affiliations:** 1 Department of Oral Health, School of Clinical Sciences, Auckland University of Technology, 90 Akoranga Drive, Northcote, Auckland, 0627 New Zealand; 2 School of Public Health and Interdisciplinary Studies, Auckland University of Technology, Auckland, New Zealand; 3 Centre for Interdisciplinary Trauma Research, Auckland University of Technology, Auckland, New Zealand

**Keywords:** Child abuse and neglect, Child protection, Child maltreatment, Oral health practitioners, Dental practitioners, Dental therapist, Knowledge and attitudes, Oral health threrapist

## Abstract

**Background:**

Child abuse and neglect are significant social and health issues in New Zealand. As the government provides free oral care to children and adolescents, oral health practitioners are positioned to respond to child protection concerns. However, research on the knowledge and attitudes of oral health practitioners is limited. This study aimed to understand the knowledge and attitudes of New Zealand dental and oral health therapists in detecting and reporting child abuse and neglect.

**Methods:**

In this descriptive exploratory cross-sectional study, we invited registered New Zealand dental and oral health therapists treating children and adolescents to the anonymous online survey.

**Results:**

Among the 92 dental and oral health therapists, 72% agreed that they could recognise the signs and symptoms of child abuse and neglect. Yet, only 48% agreed they were familiar with the reporting process. During their professional careers, 62% had at least 1 suspected case; and only 21% had ever reported their concerns. Fear of false reporting (70%) was the most significant barrier.

**Conclusions:**

Participants understood child abuse and neglect as significant social issues; however, the knowledge and attitudes to respond were limited. Efforts to enhance the knowledge and attitudes will be necessary to promote child safety and wellbeing.

## Background

Children and young people’s rights to safety and health are enshrined in the United Nations Convention on the Rights of the Child 1993. Child and adolescents’ age and developmental status mean that adults are responsible for protecting against abuse. Child abuse is any form of physical, emotional, or sexual ill-treatment, or neglect, resulting in actual or potential harm to the child’s health and development [1, 2]. Multiple studies confirm the detrimental impacts of adverse childhood experiences, including severe impairments to social development, learning, and physical and emotional health [3, 4]. Unfortunately, many New Zealand (NZ) children and adolescents are still being harmed. A cumulative prevalence analysis of NZ children born between 1998 and 2015 (55,443 children) indicated that 23.5% had at least 1 report to a child protection agency (including NZ Police and child protection agency Oranga Tamariki). Furthermore, 9.7% were confirmed as victims of child abuse and neglect (CAN) by the age of 17 years, and 3.1% had experienced an out-of-home placement [5].

The World Health Organization [6] calls for a scaling up of a comprehensive systemic and collaborative approach of violence prevention programs involving governmental and non-governmental agencies to protect children. Health professionals hold significant roles in every phase of child protection responses, from prevention to victim support. However, the roles of oral health practitioners in child protection have received little attention. The NZ public funding scheme for oral health allows children and adolescents to have free routine dental care from birth to 18 years. This scheme provides the opportunity for children to have multiple and regular interactions with oral health practitioners in school-based, community-based, or private dental clinics with or without their caregivers. Oral health is often described as a window to overall health [7]. There are orofacial manifestations of CAN that can be detected in a dental setting [1]. In a systematic review, Sarkar et al. [8] confirmed various facial and intraoral indications, including abrasions, contusions, and lacerations, as common markers of child physical abuse. Behavioural and mental health manifestations of CAN include excessive defensive or aggressive behaviour, improper sexual behaviour, and excessive fear of caregivers [9]. Lalor and McElvaney [10] found a strong relationship between child abuse experiences, particularly emotional abuse, and childhood aggressiveness, anxiety, depression, eating and attention disorders. Oral health practitioners can identify the oral and behavioural manifestations of CAN during routine check-ups and provide the necessary support to the patients. Unfortunately, no regional data is available investigating the association between CAN and dental caries. However, international research confirms the strong relationship between the 2, highlighting the critical role of oral health practitioners in child protection [11].

In NZ, preventive and restorative oral health care up to the age of 18 is mainly provided by dental therapists (DTs) or oral health therapists (OHTs) in various dental settings. DTs’ practice includes providing oral health assessment, diagnosis, and treatment focusing on dental diseases while the practice of OHTs focuses on both dental and periodontal diseases. DTs and OHTs can provide care to all age groups depending on the level of education. The NZ dental system puts oral health practitioners in a favourable position to detect CAN signs and symptoms, provide the necessary support to families, and report to appropriate child protection agencies for multi-agency response [12]. Dental visits might be the only contact with health practitioners for some children and adolescents, as regular medical visits are not routine for many.

Since reporting suspected CAN is not mandated in NZ, oral health practitioners are expected to respond to suspected CAN according to their clinical judgment and personal and professional ethical standards [13]. The Dental Council of New Zealand (DCNZ) [14–16] states that oral health practitioners should “act to protect the interests of tamariki (children), mokopuna (grandchildren), rangatahi (youth) in cases of suspected neglect or abuse by disclosing information to a relevant authority or person”. The Family Violence Act 2018 (the Act) and the escalation guideline from the Privacy Commissioner [17] provide a framework and protection for health practitioners to share information when there are child safety concerns appropriately. However, international studies suggest fewer than half of the suspected cases are reported for any further action [18–20]. The pioneering study by Tilvawala et al. [12] recognised the significance of New Zealand DTs’ role in child protection while documenting limited reporting of suspected cases to authorities by DTs. Since then, professional education has evolved, and the new profession of oral health therapy has been recognised in NZ since 2017 [21]. Furthermore, the enactment of the Act provides additional support and protection mechanisms for oral health practitioners to respond to child protection concerns. These changes mean there is more to learn about how competent and comfortable NZ oral health practitioners are in detecting and reporting potential CAN cases.

The current study set out to assess the knowledge and attitudes of both DTs and OHTs towards CAN, experiences of responding to CAN, and understanding of the impact of the Act. The study is based on the public health model of prevention for CAN, which offers a unique structure to address population-level health issues in a coordinated manner through a multi-disciplinary approach bringing evidence-based primary prevention strategies to the public [22]. The aims of this study were to 1) assess the knowledge and attitudes of NZ DTs and OHTs in detecting and reporting CAN, 2) understand potential barriers and facilitators to detecting and reporting suspected CAN, 3) investigate the impacts of the Family Violence Act 2018 on current knowledge and attitudes, and 4) evaluate perceptions on the mandatory reporting of suspected cases.

## Methods

### Study design

This descriptive exploratory survey study was conducted in NZ between June 2020 and July 2020. Registered DTs and OHTs were invited to complete an anonymous online survey. Ethics approval was gained from the [location masked for review process] Ethics Committee (Reference 20/39).

### Study samples and recruitment

Approximately 1100 DTs and OHTs are registered in NZ with a valid annual practising certificate (2019–2020). The sample frame for the current study included the 580 DTs and OHTs who are members of the New Zealand Dental and Oral Health Therapists Association (NZDOHTA), a national organization representing and advocating for DTs and OHTs in NZ. The NZDOHTA distributed emails in June 2020 containing the study information and the link to the anonymous online survey to their members.

### Data collection survey

Structured, self-administered survey data were collected using the online survey platform Qualtrics^XM^. The survey was adapted from the self-administered postal questionnaire used by Tilvawala et al. [12], with approval from the primary author. The survey introduction reiterated the purpose of the study, consent process, researchers’ contacts, and given the sensitive nature of the survey, details of a national free mental health counselling service.

The survey comprised 23 questions in 4 sections. The first section collected participant characteristics to ensure inclusion criteria were met and to assess the representativeness of the OHT and DT participants. Variables included age, gender, professional group, current annual practising certificate retention, and working experience. The second section had 4 questions assessing participant knowledge and attitudes towards detecting the signs and symptoms of CAN and reporting suspected cases. Questions included: ‘Child abuse and neglect is an important social issue in New Zealand’, ‘I can easily recognise the signs and symptoms of child abuse and neglect’, ‘I am confident in recognising the signs and symptoms of child abuse and neglect’ and ‘I am familiar with the reporting process and protocol for potential child abuse and neglect cases’. Participants were asked to select a response from a seven-point Likert scale from strongly agree (1) to strongly disagree (7), with a neutral ‘neither agree nor disagree’ (4) option.

The third survey section addressed the experiences of CAN within their practice. Firstly, they were asked to estimate the number of suspected and reported CAN cases they had encountered in the past year and during their careers. They were then asked to describe common features from suspected cases and barriers and facilitators to detecting and reporting suspected CAN. Open text fields were provided for describing common features and facilitators to detect and report CAN. In terms of barriers, 11 potential barriers were listed based on common barriers cited in the literature [12, 23]. An open text box was provided to describe any additional barriers. Two questions were asked about any postgraduate course or training attendance related to CAN.

The fourth section contained 4 questions to investigate the impact of the Act, along with personal opinions about mandatory reporting of suspected cases by health professionals. The last question provided a free text box to provide any final comments on the survey topic.

### Data management and analysis

Responses were exported to the online statistical software IBM Statistical Package for the Social Sciences (SPSS, version 26.0). Data were first inspected for completeness, followed by a review of participant characteristics to confirm meeting of inclusion criteria. Of the 580 survey invitations emailed, 112 responses were received. Twenty responses were excluded from analysis as they did not fit the inclusion criteria: a professional other than OHT or DT (4), no valid APC or not practising in New Zealand (3), no interaction with children or adolescents (1), not enough data to assess inclusion criteria (3), and no data (8). Subsequently, 92 responses (16%) were included in the final dataset.

For the final dataset, participant characteristics, including the profession, age, working experience, employment status, and the number of appointments with children and adolescents, were presented alongside NZ Dental Council Workforce analysis 2018–2019 data [24]. Descriptive analysis was conducted for all closed-ended questions. For questions where participants reported case numbers, any answers in a range were converted into mean numbers to enable further analysis. Open-ended responses were condensed into a summary format using a general inductive approach, identifying categories and providing examples for transparency [25].

## Results

### Participant demographics

Among the 92 participants,27 were DTs, and 65 were OHTs. They typically had fewer than 5 years of working experience (66%), were working in the public dental sector (55%), and routinely saw more than 25 children or adolescents each week (67%). Compared to the DTs and OHTs included in the NZ Dental Workforce 2018–19 analysis data [24], participants were younger, and OHTs were over-represented (Table 1).

**Table 1:** Participant demographics compared with the NZ Dental Council Workforce analysis 2018–2019 data (*n* = 962)

Variable	Participants *n* = 92 (%)	Workforce Analysis 2018–19 Dental and oral health therapists (*N* = 962) [24] n (%)
Profession		
OHTs	65 (70.7%)	550 (57.2%)
DTs	27 (29.3%)	412 (42.8%)
Gender		
Male	10 (10.9%)	62 (6.4%)
Female	82 (89.1%)	896 (93.1%)
Gender diverse / unspecified	–	4 (4.2%)
Age (years)		
< 25	27 (29.3%)	131 (13.6%)
25–34	34 (37.0%)	322 (33.5%)
35–44	11 (12.0%)	118 (12.3%)
≥ 45	20 (21.7%)	391 (40.6%)
Years working experience		
< 5	61 (66%)	NA
6–10	13 (14%)	
11–20	7 (8%)	
≥ 20	11 (12%)	
Employment status		
Public sector	51 (55%)	NA
Private sector	24 (26%)	
Tertiary education	1 (1%)	
Public and private	15 (16%)	
Public and tertiary education	1 (1%)	
Number of appointments with children and adolescents per week		
< 5	16 (17%)	NA
6–10	6 (7%)	
11–24	8 (9%)	
≥ 25	62 (67%)	

Note: *NA* This data is not available in Workforce Analysis 2018–19

### Knowledge and attitudes toward CAN

**Fig. 1:**
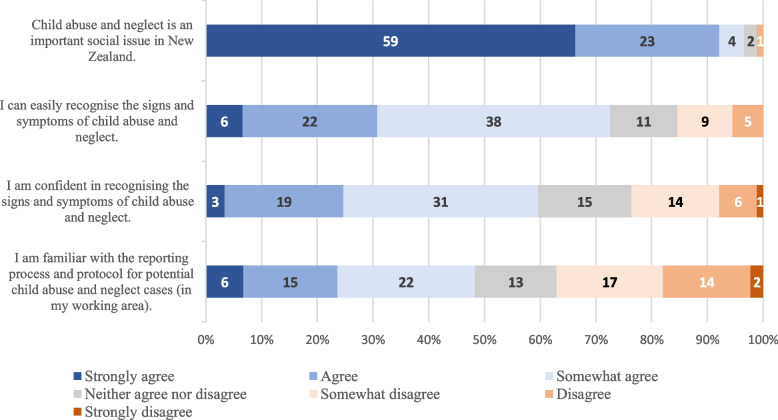
Participants’ opinions towards child abuse and neglect and self-perceiving knowledge (*n* = 89)

While CAN was strongly believed to be an important social issue in NZ, participants advised that they are ‘somewhat’ confident in recognising the signs and symptoms of CAN and ‘somewhat’ familiar with the CAN reporting process and protocol. Fig. 1 illustrates the breakdown of individual questions.

### Past experiences in detecting and reporting suspected CAN cases

Among the 84 participants who answered section 3, 74% identified 1 or more suspected cases during their careers; however, only 21% had ever reported their concerns to child protection agencies. On average, participants suspected 2 cases in the past year and seven cases during their careers.

Fifty participants noted low socioeconomic status (SES) and ethnicity as being common features fo suspected cases.

"*I believe there is a link between those in lower socioeconomic groups and of Māori and Pacific Islander ethnic groups with higher cases of suspected child abuse and neglect*".

### Potential barriers and facilitators to detect and report suspected CAN cases

**Fig. 2:**
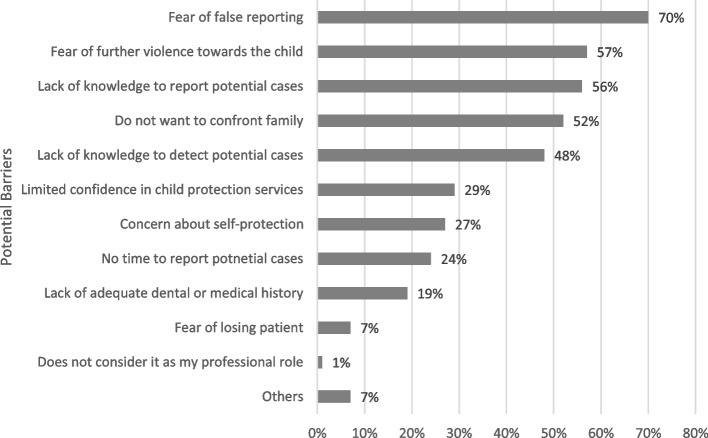
Barriers preventing participants from reporting potential child abuse and neglect cases (multiple answers allowed) (*n* = 84)

Almost all respondents (99%) considered reporting CAN cases as a part of their professional role. However, fear of false reporting (70%) was considered the most significant barrier to reporting CAN cases (Fig. 2). In addition, more than half of respondents endorsed fear of further violence towards the child, lack of knowledge to report cases, and unwillingness to confront family as barriers. ‘Other’ responses included having no barrier, inability to obtain a second opinion, and inability to maintain contact with the family.

Fifty responses described facilitators to detect and report potential CAN. Common themes included regular education and training, as well as having clear and up-to-date processes and guidelines. Training on detecting and reporting potential cases and providing interdisciplinary support would help oral health practitioners to respond more effectively.

“*Further courses and talks with speakers of other professions who deal with these types of situations such as social workers and liaising with them frequently*”.

The survey indicated that only 36% of participants had attended any course or training related to CAN after completing undergraduate training. Seventy percent of participants who had taken part in any course or training reported that it was helpful.

### Legislation relevance to oral health practitioners

There was a poor understanding of the Act, which provides additional support and protection mechanisms to health practitioners and its impact on the participants and their practices. Sixteen out of 84 responses have heard about the recent implementation of the Act, whilst only 2 participants could roughly describe the impacts on their professional practice.

The majority of participants (74%) endorsed mandatory reporting. Most participants considered reporting as a primary duty of health practitioners. Some responses recognised that DTs and OHTs might be the child’s only point of contact with a health provider to detect and report potential cases. Yet, some participants disagreed as it was perceived to limit practitioners’ autonomy. Some expressed their concerns about increased inaccurate reporting causing unnecessary trauma for children and caregivers.

## Discussion

### Knowledge and attitudes

Most DTs and OHTs participants considered CAN as significant health and social issue in NZ. Oral health practitioners hold the potential to be involved in the prevention and early intervention of CAN. However, it appears that DTs and OHTs may not be routinely reporting suspected cases. During the 2019 financial year, 82.1% of children aged 1–14 had at least 1 dental health care worker visit [26]. Because roughly 10% of NZ children and adolescents get substantiated as CAN victims before they turn 18 years old [5], oral health practitioners will likely encounter suspected CAN cases.

Even though the definition of CAN can vary among countries, oral health practitioners’ low reporting rates are observed worldwide [18–20]. In Denmark, 38% of dentists and dental hygienists suspected CAN during their careers; among those who had suspected CAN, only 1 in 3 (34%) reported their concern for further investigation [23]. The current study’s findings are also consistent with the previous NZ study of DTs conducted by Tilvawala et al. [12], which indicated 46% had identified suspected physical abuse and 40% for suspected child neglect; however, 29 and 22% reported, respectively.

Low SES was mentioned most as an observed common feature for suspected CAN patients. Various studies have noted a relationship between the socioeconomically disadvantaged and CAN [27, 28]. However, the association must be interpreted with caution, as socioeconomically disadvantaged families experience poverty, housing and food insecurity, and social and health inequalities, which often lead to parental depression and substance abuse. These conditions can contribute to abusive and neglectful behaviours. Apart from parental depression and substance abuse, other factors can diminish caregivers’ abilities.

Participants also identified CAN over-representation for Māori and Pacific Island children. The literature has indicated that identifying with particular ethnic and social groups can increase the likelihood of child protection concerns being detected and reported [29]. In the NZ context, understanding the impact of colonisation and social and institutional racism on health and social inequalities is necessary to respond effectively [30, 31]. Stereotyping can cause over-diagnosis for Indigenous Māori and Pacific Islander children and children from families with low SES [29]. Providing family violence prevention and early intervention designed to suit Indigenous Māori and Pacific Islander families is most likely to achieve the best outcome for children and families [31]. On the other hand, children from privileged families may receive less attention, with health practitioners missing the signs and symptoms of abuse and neglect and the opportunity to intervene. Further research is critical to understand how the pre-existing perception of DTs and OHTs towards patients with different SES affects their attitudes in the detection and reporting of CAN. Importantly, understanding the close association between intimate partner violence (IPV) and CAN will be beneficial to understanding the broad picture of family violence. Both IPV and CAN are different forms of family violence with shared risk factors that occur concurrently in a family [32]. Increasing the understanding of IPV and its impacts on the child’s health and the potential harm can enable an integrated and effective response to victims and their families and contribute to a prevention and early intervention approach.

DTs and OHTs reported 2 dominant barriers to detecting and reporting CAN: 1) fear of causing harm to the patient and 2) a lack of knowledge to detect and report. The barriers indicate a necessity to improve oral health practitioners’ knowledge of child protection. Even though identifying oral manifestations of CAN and reporting procedures are part of undergraduate training, knowledge gaps and lack of confidence are evident among DTs and OHTs. These findings are consistent with other international studies. The fear of false reporting and further violence has been reported in many studies, including the NZ study by Tilvawala et al. [12] (69% fear of false reporting), UK study by Harris et al. [33] (78% lack of certainty about diagnosis, 53% fear of family violence), and Scotland study by Cairns et al. [18] (88% uncertain about the diagnosis, 34% fear of family violence). Similarly, lack of knowledge was commonly reported elsewhere by Harris et al. [33] (32% lack knowledge of referral procedures) and Cairns et al. [18] (71% lack knowledge of referral procedures).

More than half (52%) of the current research participants were unwilling to confront families of potential victims. This behaviour may be linked with concerns about self-protection, confidentiality, or time restrictions. This study did not investigate participants’ more profound understanding of how those barriers have formed and how they influence current responses to CAN, which needs further attention. An increased understanding of the current low responsiveness toward CAN will help oral health professionals to enhance their knowledge and attitudes.

In the current study, participants provided potential facilitators to help DTs and OHTs in child protection. Seventy percent who attended courses or training found them beneficial. Responses to open-ended questions support the effectiveness and potential benefits of having child protection training to gain up-to-date information on reporting pathways and policies and connect with other health and social professionals to work as an interdisciplinary team. The New Zealand Dental Association [13] guideline assists oral health practitioners with child protection and guides practitioners’ responsibilities. However, the guideline is from a professional dental association rather than a regulatory authority. DTs and OHTs usually do not belong to the New Zealand Dental Association which focuses on advocating for dentists and dental specialists, therefore, DTs and OHTs are unlikely to read the guideline. The study findings have implications for developing a comprehensive guideline that can be incorporated into the DCNZ professional standards framework and tertiary training programs. In a qualitative meta-synthesis, Hegarty et al. [34] identified collaboration among health and social practitioners and being supported by the health system as the main themes to improve the readiness of health practitioners to address family violence. Evidence indicates that the multidisciplinary team approach is more effective in improving responses than stand-alone practices. Designing a training programme that guide practitioners to access multidisciplinary support and embedding this in the professional standards framework and tertiary education programmes would be essential. The actual availability and effectiveness of any child protection courses and training were not examined in this study, which might provide a better understanding of the practitioner’s training needs. Future studies on the current topic are therefore recommended.

Another issue highlighted was participants’ limited awareness of the Family Violence Act 2018 and its impact on their practices. The Act provides support and protection mechanisms, including 1) participants’ abilities to request, use, or disclose personal information for purposes related to CAN, 2) what to consider when disclosing personal information, and 3) necessary protection that participants can access when disclosing information. A majority of the participants were not aware of the Act, indicating a potential communication gap among the government, the professional and regulatory bodies and the frontline oral health practitioners. Providing accurate information on how the government can provide the necessary support has the potential to act as a facilitator to support both potential victims and practitioners by supporting oral health practitioners to detect and report potential cases more confidently.

Most participants (74%) agreed with mandatory reporting of suspected cases; however, there is an ongoing debate regarding its effectiveness [35]. A mandatory reporting system may create a culture among health practitioners to report frequently within the legal boundaries. However, there are obvious barriers to implementing mandatory reporting, including health practitioners’ resistance and having no gold standard to diagnose and identify potential cases. Most importantly, lack of knowledge to adequately detect and report suspected cases would prevent the implementation of mandatory reporting as the mandatory reporting system would not work if practitioners do not know how to respond with the situation [36]. Further investigation to assess the feasibility and efficiency of a mandatory reporting system would be necessary.

### Strengths and limitations

A strength of the current study is that it included both dental and oral health therapy professions, which together provide most children with oral health care in various community settings. As a result, the participants had a high rate of involvement in children and adolescent oral health care. The findings reinforce a strong need to improve the knowledge and attitudes of DTs and OHTs for the future generations of the 2 professions.

This study’s limitations include the relatively low response rate (16%) and a greater representation of oral health therapists than dental therapists (Table 1). OHTs are a more recently established profession; therefore, most OHTs had their undergraduate training within the last 10 years, while most DTs would have had more clinical experiences. It is unclear how the 2 professions would respond differently to CAN in their practice. Additionally, there could be differences between those who responded, and those who did not. As the survey was sent out by email, it may have increased the accessibility and responsiveness to younger practitioners who are represented more in the oral health therapy workforce. It may be that non-respondents were either more or less likely to be engaged with child protection practices. The COVID-19 pandemic may have also impacted the response rate, as the survey was sent out soon after NZ’s first national lockdown, where the profession was focused on adapting to the new COVID regulations and practice standards. Given the low response rate, the outcome cannot be necessarily considered as wholly representative of NZ dental and oral health therapy professions. The focus of the study is to understand the knowledge and attitudes of DTs and OHTs in detecting and reporting child abuse and neglect, however, to fully understand the whole oral health profession, dentists and paediatric dentists could have been invited as some adolescents are also examined and treated by them.

In terms of the questionnaire, even though the study adopted a previously developed questionnaire and was further piloted by 2 DTs and 2 OHTs, it was not fully validated to evaluate the structure of the survey. Some questions in the second part of the survey generated skewed results as it is hard to disagree that child protection is an important social issue. Also, several questions included both child abuse and child neglect, however, signs of symptoms of the 2 issues are different [9]. Asking specific questions on each issue would have provided further understanding of participants’ knowledge and attitudes. Another limitation was that competence in recognition of CAN was self-reported. Participants may have under- or over-recognised CAN cases, however, due to the limitation of the survey research, it was not possible to assess the accuracy of their reporting. Furthermore, some participants provided ranges for case numbers rather than a specific number which were converted into means. Using means increased the risks of losing outliers and underestimating the variance of responses.

### Implications for public health and future research

A CAN identification process is highly reliant on health practitioners’ personal judgment and a clear understanding of their roles and responsibilities [37]. This emphasises the need to improve oral health practitioners’ understanding in detecting CAN cases in early stages, provide the necessary support to children and their families, and report to the child protection agencies to provide safer environments to children and adolescents in need. Despite challenges to measuring the impact of early intervention approaches on child protection, McCarry et al. [38] identified a perceived need and positive impact of the early interventions approach by children, mothers, and service providers to effectively safeguard children from family violence. Emphasising the need for evidence-based early intervention approach to prevent further harm to the child is equally crucial to detecting and reporting potentially imminent harm. The participants’ desire to improve their knowledge and attitudes toward child protection is promising. The consensus statement on future directions for the behavioural and social sciences in oral health research [39] emphasises the need to address social and environmental determinants. Further study will be required to explore how proximal determinants are affecting the responsiveness of oral health practitioners to CAN and those factors can be addressed to improve practitioners’ responses. Understanding those determinants can enhance child’s safety and wellbeing by improving the responsiveness of oral health practitioners and facilitating early-intervention approaches to child protection.

Further research will be necessary to include other oral health practitioners such as paediatric dentists and community dentists, other relevant stakeholders, and community members to share their perspectives on CAN and the role of oral health practitioners in child protection. Investigating the impediments and associated impacts on the responsiveness of oral health practitioners to children’s safety and wellbeing needs would be required. Key findings can be translated into oral health practitioners’ early intervention approaches to child protection to achieve child safety and wellbeing.

Given the complexity of family violence, it is unlikely that a single guideline will suffice [37]. Even so, having practical guidance from the regulatory authority can increase oral health practitioners’ confidence to take action. The guideline shouldbe easily accessible by practitioners and regularly updated to ensure current and relevant information. As oral health services are often provided in a school setting, information should incorporate an interprofessional approach to communicate with other health, education, and social professionals and share knowledge with each other. The guideline should provide information on how DTs and OHTs can support the family and the community, not just detecting and reporting the potential CAN cases. Providing necessary support helps as a part of holistic care to the family and the community across the continuum of needs that can protect children and adolescents from further harm from CAN [38]. The government recently introduced Te Aorerekura (the national strategy to eliminate family violence and sexual violence), which includes a reformation of NZ healthcare to make it more equitable and better suited to meet the needs of all people [40]. This will be a crucial moment to review oral health practitioners’ roles in child protection practices. Further consultations with DTs and OHTs will be required to provide ideal support.

Carefully designed courses to educate DTs and OHTs to improve understanding, knowledge, and attitude are required to improve responsiveness to child protection to detect suspected CAN cases and provide adequate support to affected children and families. Even for health professionals who have extensive prior experience in dealing with CAN cases, consistent engagement with continuing developments and training is beneficial to maintain a capability to detect and report suspected cases. The course should train practitioners to be able to access necessary resources when needed, seek professional advice from other health or social practitioners, and approach multi-disciplinary team support. The focus of the training should be based on the needs of oral health practitioners, stakeholders, communities, and service users. Additionally, this should be a valuable opportunity to engage with Māori and Pacific communities to understand structural racism in health and child protection practices and address those inequity issues in oral health practices in NZ.

Further investigation to understand the reasons for the under-reporting of child protection concerns by DTs and OHTs is required and should be addressed at both individual and professional levels. Currently, there are insufficient courses related to CAN available for oral health practitioners in NZ. Stakeholders should work collaboratively to design appropriate courses that can be delivered regularly, ensuring the educational material is readily accessible to all oral health practitioners.

## Conclusion

This exploratory research showed insufficient understanding of participating dental and oral health therapists in responding to CAN. While practitioners perceived positively their ability to detect and report suspected cases, the actual detection and reporting rates were considerably low. Insufficient knowledge needed to detect and report suspected cases and fear of false reporting were identified as major barriers. The outcome of the study indicates the importance of improving the knowledge and attitudes of DTs and OHTs to protect children and adolescents from CAN. Oral health practitioners should consider participating in ongoing training to enhance competency to prevent and respond to CAN. The formation of a comprehensive national guideline and an interdisciplinary approach can be considered to assist oral health practitioners.

## Data Availability

The datasets used and/or analysed during the current study are available from the corresponding author on reasonable request.

## Abbreviations

CAN: child abuse and neglect
DTs: dental therapists
IPV: intimate partner violence
NZ: New Zealand
NZDOHTA: New Zealand Dental and Oral Health Therapists Association
OHTs: oral health therapists
The Act: the Family Violence Act 2018
